# Adolescent Crohn’s disease: A nursing case report

**DOI:** 10.1097/MD.0000000000046251

**Published:** 2025-11-28

**Authors:** Xiaoran Wang, Nan Zhang, Hongwen Ma

**Affiliations:** a Department of Gastroenterology, Tianjin Union Medical Center, The First Affiliated Hospital of Nankai University, Tianjin, China; b Department of Nursing, Tianjin Union Medical Center, The First Affiliated Hospital of Nankai University, Tianjin, China.

**Keywords:** adolescent, Crohn disease, exclusive enteral nutrition, infliximab, nursing care

## Abstract

**Rationale:**

Crohn disease (CD) is a chronic, relapsing gastrointestinal tract disorder. The incidence of CD is increasing worldwide. Comprehensive nursing care is essential for managing complex treatment regimens and promoting adherence in this population. While biologics and exclusive enteral nutrition (EEN) are established treatments, nursing-led strategies to ensure adherence and manage the transition between these therapies are poorly documented, especially in the home-care setting.

**Patient concerns:**

An adolescent patient with a recent history of perianal abscess surgery presented with intermittent abdominal pain, fever, poor wound healing, unformed stool, loss of appetite, and significant weight loss. The patient later developed a suspected enterocutaneous fistula, leading to intensified symptoms and emotional distress.

**Diagnoses:**

The patient was diagnosed with CD, accompanied by mild anemia, gastrointestinal dysfunction, intestinal flora imbalance, and nutritional risk. A suspected small intestinal fistula was identified during follow-up.

**Interventions:**

Critical nursing care includes biological agent care, nutritional support, pain control, psychological care, skin care, observation and nursing care of fever and pain, and home health guidance.

**Outcomes:**

Through nursing interventions, the patient’s clinical condition significantly improved. He achieved clinical remission, evidenced by the resolution of fever and abdominal pain, improved inflammatory markers, and a weight gain of 3 kg. Notably, the patient successfully adhered to the prolonged out-of-hospital EEN and subsequently transitioned back to a normal diet.

**Lessons:**

This case highlights the critical role of individualized and sustained nursing care in improving clinical and psychological outcomes in adolescents with complex CD. Integrating advanced biologic treatment with EEN under close nursing supervision proved feasible and effective. These findings support the adoption of a holistic nursing approach that addresses both physical and emotional needs in similar cases. Further research should focus on standardizing integrative nursing protocols for pediatric inflammatory bowel disease.

## 1. Introduction

Crohn disease (CD) is a chronic, relapsing gastrointestinal tract disorder.^[1]^ Consequently, the incidence of CD is increasing globally, particularly in industrialized countries with increasingly serious air pollution.^[2]^ The pathogenesis of CD is unclear. However, it is widely recognized that the complex interaction between genes, environment, and immunity causes it.^[3]^ Abnormal immune responses to gut bacteria in genetically susceptible hosts are thought to underlie the disease. Abnormalities in gut bacteria or their products can also trigger innate and adaptive mucosal immune responses in the host, exacerbating intestinal immune damage.^[4]^ However, the incidence of CD in adolescents is increasing. Thus, adolescent CD patients need to pay extra attention to growth and development and face more significant difficulties in treatment and management. Biologic therapies, sometimes called biologics, are a class of medications that reduce intestinal inflammation by targeting specific immune system responses. Two classes of biological agents are used to control CD symptoms: anti-TNF agents and anti-integrin medications. Notably, these medications are often given long-term, sometimes along with immunomodulators.^[5]^ Exclusive enteral nutrition (EEN) is best described as providing nutritionally complete liquid formula, without exposure to other foods, to patients with CD, usually for 6 to 8 weeks. Its success in inducing clinical remission and intestinal mucosal healing in a high proportion of patients with active inflammatory CD represents a significant paradigm shift in approach. Unfortunately, CD symptoms affect the psychological well-being of children by affecting their social confidence.^[6]^ Several treatment pathways for remission induction in pediatric CD are available, with EEN as the recommended first-line therapy.^[7]^

## 2. Case presentation

### 2.1. General information

Patient, male, 15 years old, complained of intermittent abdominal pain and fever over a year. The patient had no family history of inflammatory bowel disease or other autoimmune disorders. His medical history began on January 7, 2022, when he was admitted to the anorectal surgery department for a perianal abscess. The abscess was resected under spinal anesthesia on January 10, 2022. However, the patient had poor wound healing, unformed stool, intermittent fever, the highest body temperature of 37.5°C, loss of appetite, a 1/3 reduction in food intake and a weight loss of 7 kg. On admission, laboratory investigations revealed markedly elevated inflammatory markers, with a C-reactive protein level of 46.12 mg/L and an erythrocyte sedimentation rate of 23 mm/h. Moreover, physical examination showed poor nutritional status: total protein 64.1 g/L, albumin 31.5 g/L, and a hemoglobin concentration of 98.0 g/L, with a weight of 70 kg and a body mass index of 22.09. A colonoscopy showed inflammatory changes and inflammatory bowel disease, and subsequent histopathological examination of biopsy specimens confirmed active inflammation. The findings included chronic mucosal inflammation with focal ulceration, a mixed inflammatory infiltrate composed of neutrophils, plasma cells, and lymphocytes, lymphoid hyperplasia, and crypt abscesses with neutrophil invasion of the glandular epithelium. CT imaging identified diffuse inflammatory wall thickening affecting the terminal ileum, right colon, mid-sigmoid colon, and appendix. Combined with the results of colonoscopy and abdominal CT following admission, the patient was diagnosed with CD (A1bL3bB1p), mild anemia, gastrointestinal dysfunction, intestinal flora imbalance, and nutritional risk. The patient expressed significant anxiety upon receiving his diagnosis, fearing the impact on his schooling and social life.

### 2.2. Treatment and outcome

The doctor’s treatment plan was to choose biological agents supplemented by mesalazine (4 g/d), oral flora regulation, enteral nutrition, symptomatic support, etc. Simultaneously, patients should be provided high-calorie, high-protein, vitamin-rich, easy-to-digest foods while maintaining the balance of water, electrolytes, and acid–base. The patient completed biological agent therapy and began regular 8-week maintenance infusions. However, the patient had a sudden onset of right lower abdominal pain, high fever, and suspected small intestinal fistula formation on December 28, 2022. During this time, anti-inflammatory, nutrition, and fluid replacement therapy were administered. Following admission, the patient presented with weight loss, anemia, malnutrition, balance disorders of water, electrolyte and acid–base, and emotional changes. The patient received enteral nutrition support, pain care, and psychological care. Still, the symptoms were not significantly relieved. Thus, the doctor suggested surgical treatment, but the patient refused. Therefore, the doctor adjusted the treatment in February 2023, replaced anti-inflammatory drugs, and gave the patient EEN and fluid infusion treatment. Subsequently, the patient was discharged and received out-of-hospital EEN for 3 months with close nursing monitoring and support to ensure adherence. This nursing-led support was critical to the success of the regimen. By May 2023, the patient’s vital signs were normal, the inflammation was improved, the abdominal pain was relieved, the condition was stable, and the weight increased by 3 kg. The patient’s mood and psychological well-being also improved significantly throughout this period, supported by continuous nursing care.

## 3. Nursing

### 3.1. Biological agent care

The patient was treated with infliximab (IFX), a biologic agent. IFX is suitable for CD, ulcerative colitis, and rheumatoid arthritis. Still, it is contraindicated in patients who are allergic to murine protein, have tuberculosis or other active infections, and have moderate to severe heart failure. Generally, the first dose is 5 mg/kg, and the same dose is given on the second and sixth week and then every 8 weeks following administration.^[8]^ The doctor should still adjust the dosage according to the patient’s condition. For adult CD with poor efficacy, 10 mg/kg/ time can be considered, and the drug administration time should not be <2 hours. And IFX may increase the risk of serious infection and should not be used in active patients. If jaundice occurs, the drug should be stopped.

Prior to each infusion, a rigorous assessment was conducted to screen for contraindications, particularly active infections. Vital signs were baseline documented. We meticulously reconstituted the drug according to the manufacturer’s guidelines and administered it via an infusion pump to ensure absolute accuracy in rate control. IFX infusion speed was strict and observed the specific speed order: 15 mL/h for 15 minutes, 20 mL/h for 15 minutes, 40 mL/h for 15 minutes, 80 mL/h for 15 minutes, 150 mL/h for 30 minutes, and finally 250 mL/h until completion. The patient was placed on continuous cardiac monitoring throughout the process. We remained at the bedside for the first critical 15 minutes and conducted assessments every 15 to 30 minutes thereafter to monitor for acute adverse reactions such as fever, chills, dyspnea, or hypotension. We also observed the patient for adverse reactions and instructed the patient and family members to notify the medical team when the discomfort was noted. At this time, of course, we suspended the infusion of other drugs to ensure that incompatibility did not occur. IFX infusion may cause adverse reactions, such as infection, allergic respiratory symptoms, gastrointestinal reactions, dizziness, palpitation, and other abnormal reactions. The patient and his family were educated on the contraindications of IFX use, common adverse reactions, and precautions.

### 3.2. Nutritional support

Nutrition is essential in the treatment of the patient. The advantage of nutritional support is that it has no side effects, can induce disease remission, and promotes intestinal and enterocutaneous fistula healing.^[9]^ Moreover, it can prevent the patient from undergoing surgery when combined with antibiotics. When the patient did not respond well to biological agents and refused surgery, he chose EEN, which is the strictest diet plan and the most effective method to induce remission of CD.^[10]^ During hospitalization, the patient had many nutritional problems, such as emaciation, anemia, malnutrition, and fluid and electrolyte imbalance. During this time, nutritional care for patients is critical.

For the patient, we evaluated his nutritional status. We also told the patient or his families about nutrition knowledge, instructing them to regularly monitor body weight, supplement enteral nutrition on time, and administer the EEN strictly. In addition, the patient was advised to develop good living habits, eat on time, pay attention to rest, maintain a positive state of mind, and have regular follow-up visits. If uncomfortable, immediately seek medical advice.

For the patient with EEN, it is more challenging to improve his therapy compliance. Our nursing focus shifted to achieving the exceptionally high level of compliance required for success, particularly challenging for an adolescent. The patient was a student, and nutritional nursing guidance was for the patient and the family members. To understand the social and psychological burden of a liquid-only diet, we engaged in motivational interviewing. We explained the importance of EEN to the patient and family members first, helped the patient and family members understand the ingredients of EEN, and relieved the patient’s concern about insufficient nutrition supply. To help the patient choose the appropriate EEN preparation combined with doctor’s advice and patient’s preference, the patient was instructed to take regular supplements and develop good eating habits.

We established a weekly telephone follow-up to troubleshoot challenges like taste fatigue, bloating, and the urge to eat solid food. We encouraged the use of a journal to track intake and symptoms. Following 3 months of EEN, the patient’s condition recovered, and his 3 kg weight gain became a powerful motivator, visually reinforcing the benefits of his perseverance. Thus, the nurse instructed the patient to take liquid food, such as rice soup, lean meat soup, and milk, while slowing the eating speed and increasing the meal time. The principle of diet is from less to more. Likewise, the family members of the patients were instructed to observe whether the patients had abdominal distension, vomiting, gastric retention, and other symptoms. If there were no repeated symptoms, the patients increased their diet according to the situation. Following this plan, the patient achieved a completely normal diet. During treatment, the patient described the EEN regimen as “the most difficult challenge,” citing taste fatigue and social isolation. However, he stated, “The nurses explained everything step-by-step and encouraged me. Knowing what to expect and having my family involved made it possible to stick with the diet.” He reported high satisfaction with the psychological support received, which he felt was crucial to his recovery.

### 3.3. Psychological care

This patient was in the growth period and may have had various psychological sensitivities.^[11]^ The patient initially presented with some anxiety and depression. Thus, the patient, who was at moderate risk, underwent psychological screening, the generalized anxiety disorder-7 scale for anxiety assessment, and the patient health questionnaire-9 for depression screening, with initial scores of 10 (moderate anxiety) and 12 (moderate depression), respectively. Recognizing the profound psychological impact of a chronic illness on an adolescent, we implemented proactive, quantifiable psychological support. We gave patients individualized psychological guidance in different periods. When the patient was first diagnosed with CD, nurses focused on explaining the diagnosis of CD, the detailed treatment methods, and the necessity of treatment to relieve patients’ anxiety and depression. When patients worried about adverse reactions when using IFX to treat diseases, nurses patiently explained the indications and contraindications of IFX, monitored the vital signs, and took the successful infusion of patients as an example to alleviate the patient’s anxiety. Nurses would also help explain the treatment plan adjustment when the IFX treatment effect was not good. When the patient used EEN, we had encouraged him to adhere to EEN, increasing his confidence in overcoming the disease. Throughout the process, we paid close attention to the patient’s mood. We encouraged him to read books and listen to music to relieve tension, improve his mental state, and alleviate his confusion. At discharge, his generalized anxiety disorder-7 and patient health questionnaire-9 scores had decreased to 5 and 6, respectively, indicating mild symptoms.

### 3.4. Skincare

Diarrhea is the primary clinical manifestation of CD. After diarrhea, the perianal skin is moist, coupled with feces, which easily stimulates the perianal skin and the repeated friction of the perianal skin. As such, these can easily cause redness and ulceration of the perianal skin, and in severe cases, they can easily cause infection. Recurrent diarrhea increases the physical and mental pain of patients and affects their emotions. Active and effective skincare is essential. During the whole process of the patient’s treatment for CD, the patient had the complication of a small intestinal fistula, and the patient had previously undergone perianal abscess surgery. Of course, attention should be paid to the perianal skin during the nursing process, and special care should also be given to the perianal skin of CD patients with diarrhea symptoms. Therefore, the patient was instructed to scrub with warm water following each defecation, avoid using irritating soap and washing supplies, change underwear frequently, and keep perianal skin clean and dry to prevent infection. Patients can follow the doctor’s advice to use the necessary ointment. The patient was also encouraged to adhere to a sitz bath.^[12]^

### 3.5. Fever and pain management

The patient had a high fever on admission and was physically cooled, but the patient’s body temperature did not decrease significantly. Following oral metronidazole antiinfective therapy, according to the doctor’s advice, the patient’s body temperature improved after monitoring. When a patient has a high fever, the patient’s vital signs are monitored. A timely symptomatic treatment was performed, and the patient’s clothing was changed frequently to avoid the cold after a patient with high fever and sweating. Ventilation was provided for 30 minutes every day to prevent indoor cross-infection. When patients have a high fever, nutritional support should be done in time, and enteral and parenteral nutrition should be used to increase nutrition. Water should also be replenished to reduce water and electrolyte loss. At the same time, oral cleanliness should be maintained to prevent infection.

The patient’s pain was assessed as 3/10 on the numerical rating scale. Nurses explained the pathophysiology of the pain etiology, counseling the patient to minimize exposure to identified precipitating factors and advise explicitly against strenuous physical exertion and Valsalva maneuvers during defecation. Furthermore, nurses provided non-pharmacological interventions, including instruction in distraction techniques, diaphragmatic breathing, and progressive muscle relaxation. His pain was effectively managed with the above interventions.

### 3.6. Home health guidance

During the treatment process, the nurses also provided the following health advice: Guide the patient in appropriate activities and rest, for example, engaging in moderate exercise and avoiding overwork; Psychological guidance was given to patients. He is advised to maintain a good attitude, which is essential to the recovery of the disease; Dietary and life guidance was given to patients. He is advised to adopt a balanced diet and lifestyle; and It is suggested that the patient should seek medical advice in time if he feels unwell during his discharge.

## 4. Discussion

This case report delineates the successful management of an adolescent male with moderate CD complicated by a perianal fistula, through an integrated medical and nursing approach centered on biologic therapy and EEN subsequently. Biological drugs and, more specifically, anti-TNF drugs such as infliximab and adalimumab have proven efficient for the treatment of IBD in adults and children.^[13]^ Notably, using biological drugs has improved pediatric inflammatory bowel disease outcomes, especially for adolescents.^[14]^ EEN has been recommended as the first-line treatment for CD-induced remission in adolescents.^[15]^ EEN can also induce CD remission without adverse effects. A randomized controlled trial included 19 children with CD, and the mucosal healing rate in EEN was 89%.^[16]^ The initial treatment with IFX was consistent with international guidelines recommending anti-TNF agents for pediatric CD. Our experience underscores the critical nursing role in ensuring safety through protocol adherence, vigilant monitoring, and patient education. Following the subsequent complication of a fistula and the patient’s refusal of surgery, EEN was implemented as a rescue therapy. However, improving the compliance of CD patients with long-term EEN treatment is a clinical problem that needs to be addressed. Early enteral nutrition is a way to help patients recover and is particularly important for their recovery. In the later course of treatment, the patient received EEN for 3 months, and the overall effect was positive. Nursing care was critical in ensuring both treatment efficacy and patient compliance, culminating in a successful nonsurgical outcome.

Studies have shown that CD patients are more prone to psychological problems, such as anxiety or depression, than the general population.^[17]^ In adolescents with CD, unpredictable disease onset, intestinal symptoms, complex treatment protocols, and therapeutic side effects cause significant distress to adolescents and parents.^[18]^ Unfortunately, the psychological factors of CD patients are often ignored in clinical practice. Considering that the patient is an adolescent, different from the psychological characteristics of adults, adolescent psychology is more sensitive and requires unique and intentional psychological care. According to the actual situation of patients, patients should be guided to establish confidence in treatment, provide adequate care, and alleviate their low mood and poor mental state. In doing so, patients can actively invest in treatment and recover as soon as possible. Importantly, care and psychological counseling can improve the mood of patients. The guidance of the nursing team and the aforementioned nursing measures ensure remission of the current patient’s condition. It also provides a basis for the patient’s smooth recovery and return to normal life. Under careful nurse guidance, patients improved their compliance and were more cooperative with the treatment. Ultimately, the patient was satisfied with the overall care of the treatment, forming the basis for the desired treatment results. This case powerfully illustrates that the efficacy of EEN is contingent upon effective nursing support that addresses the psychology and compliance.

## 5. Conclusion

This case report details the successful management of an adolescent with complex CD through biological therapy and EEN. While the medical treatments were pivotal, the critical differentiating factor was the implementation of comprehensive, individualized, and proactive nursing care. These effective nursing interventions included pain management, psychological care, observation and care of body temperature, and nutritional support. Furthermore, patient adherence is crucial. Though effective management, the patient avoided surgery, achieved clinical remission, and both her nutritional status and psychological condition were improved. The limitation of this report is its relatively short follow-up period, which precludes any assessment of long-term outcomes such as remission sustainability or the need for subsequent treatment. Larger, prospective studies are warranted to quantify the impact of such intensive, multi-dimensional nursing interventions on long-term health outcomes and quality of life in this vulnerable population.

## Author contributions

**Data curation:** Xiaoran Wang, Nan Zhang.

**Formal analysis:** Xiaoran Wang, Nan Zhang.

**Funding acquisition:** Xiaoran Wang.

**Methodology:** Nan Zhang.

**Project administration:** Hongwen Ma.

**Supervision:** Hongwen Ma.

**Writing – original draft:** Xiaoran Wang.

**Writing – review & editing:** Nan Zhang, Hongwen Ma.
